# A community based seroprevalence of SARS‐CoV‐2 antibodies in Somali Region, Eastern Ethiopia

**DOI:** 10.1002/iid3.1148

**Published:** 2024-01-10

**Authors:** Solomon Yared, Tsegalem Abera, Seid Mohammed Ali, Abdifatah Muktar Muhummed, Mohammed Ibrahim, Abdullahi Hassan, Jan Hattendorf, Jakob Zinsstag, Rea Tschopp

**Affiliations:** ^1^ Department of Biology, College of Natural and Computational Sciences Jigjiga University Jigjiga Ethiopia; ^2^ Department of Veterinary Microbiology and Public Health, College of Veterinary Medicine Jigjiga University Jigjiga Ethiopia; ^3^ Department of Animal and Range Sciences, College of Dryland Agriculture Jigjiga University Jigjiga Ethiopia; ^4^ Department of Epidemiology and Public Health, Swiss Tropical and Public Health Institute University of Basel Basel Switzerland; ^5^ School of Medicine, College of Medicine and Health Sciences Jigjiga University Jigjiga Ethiopia; ^6^ One Health Unit Armauer Hansen Research Institute Addis Ababa Ethiopia

**Keywords:** community, SARS‐CoV‐2, seroprevalence, Somali Region

## Abstract

**Background:**

Coronavirus disease 19 (COVID‐19) is life‐threatening infectious disease caused by SARS‐CoV‐2 virus that caused a global pandemic. SARS‐CoV‐2 has been widely transmitted throughout Ethiopia, with over 501,060 cases confirmed and 7574 deaths until November 2023. This study assessed for the first time the seroprevalence SARS‐CoV‐2 in the general population of the Somali Region during the COVID‐19 pandemic.

**Methods:**

A cross‐sectional study design was conducted from May to June 2021 in 14 districts of Somali Region. Blood samples were collected in 820 participants in addition to administering a questionnaire that included sociodemographic characteristics and history of clinical symptoms of COVID‐19. Blood samples were tested for the presence or absence of anti‐SARS‐CoV‐2 using a commercial Enzyme‐Linked Immunosorbent Assay (ELISA) kit (Euroimmun).

**Results:**

Overall, 477 (58.2%) were male and 343 (41.8%) were female. The majority of the participants (*N* = 581; 70.9%) were between 18 and 34 years old and not vaccinated against COVID‐19 (*N* = 793; 96.7%). The overall seroprevalence of SARS‐CoV‐2 antibodies was 41.7% (95% CI: 33.3%–47.6%). The highest prevalence was found in Goljano district (70%) and the lowest in Gunagado district (22.5%). Only age was found to be associated with COVID‐19 seropositivity.

**Conclusion:**

Prevalence of SARS‐CoV‐2 antibodies was the highest ever reported in Ethiopia, indicating that a large proportion of the population had been infected 14 months after the start of the outbreak in the country. Such studies are important to swiftly reassess and improve specific COVID‐19 preventive and control measures to reduce transmissions within the community in a given setting.

## Graphical abstract

We conducted a cross‐sectional study to estimate the seroprevalence of antibodies to SARS‐CoV‐2 in 820 participants from the general population of SRS during the COVID‐19 pandemic and risk factors for disease and disease transmission The overall seroprevalence of COVID‐19 was 41.7% (95% CI: 33.3%–47.6%). The highest prevalence was found in Goljano district (70%) and the lowest in Gunagado district (22.5%). Only age was found to be significantly associated with COVID‐19 seropositivity. The high prevalence of COVID‐19 combined with the low vaccination rate indicated that a large proportion of the population in SRS had been infected since the beginning of 2020 The key findings from the present study will have implications for shaping future epidemic control.

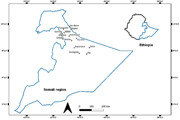

## 1 BACKGROUND

In December 2019, adults in Wuhan, capital city of Hubei province started presenting to local hospitals with severe pneumonia of unknown cause. This rapidly spread from Wuhan to other areas. On December 31, 2019, China informed the World Health Organization (WHO) about the case. On 7th January the virus was identified as a coronavirus that had >95% homology with the bat coronavirus and >70% similarity with the SARS‐CoV.^1^ The virus classified within the same species as SARS‐CoV and was named SARS‐CoV‐2 by the International Committee on Taxonomy of Viruses (ICTV).^2^ Simultaneously, WHO named the disease as COVID‐19, for “coronavirus disease 2019”^3^ and it was declared as a global pandemic on March 11, 2020. Globally, as of November 8, 2023, there have been 771,820,937 confirmed cases of COVID‐19, including 6,978,175 deaths, reported to WHO. As of November 4, 2023, a total of 13,534,474,309 vaccine doses have been administered.^4^

The first case of COVID‐19 in Ethiopia was recorded on March 13, 2020, from a 48‐year‐old Japanese national presenting himself at a public health center in the capital city, Addis Ababa a week after entering the country from Burkina Faso.^5^ Since then, more than 501,060 confirmed cases and 7574 deaths were reported in the country as of November 2023. As of May 27, 2023, a total of 68,856,793 vaccine doses have been administered.^4^

The true extent of the COVID‐19 epidemic in the world is still uncertain using current surveillance methods, which is largely limited to reverse transcription polymerase chain reaction (RT‐PCR). The reported confirmed cases likely represent only a fraction of all the infections that have occurred so far.^5^ The number of confirmed cases detected and reported in each country is influenced by many factors including limited access and/or utilization of healthcare and COVID‐19 testing, limited surveillance, lack of knowledge among the population about when to seek medical attention, and limited laboratory capacity.^6^

Moreover, there has been substantial evidence that approximately 40% of all SARS‐CoV‐2 infections are thought to be asymptomatic, and active surveillance for infections without symptoms is limited even now, large number of cases might be unnoticed/might go undocumented.^7^, ^8^ Cognizant of this fact, several large population‐based seroprevalence studies have been conducted in COVID‐19 hotspots in Europe,^9^, ^10^, ^11^ America,^12^, ^13^ Asia,^14^, ^15^, ^16^, ^17^ and in Africa.^18^, ^19^, ^20^

Population‐based serological data are essential for understanding the overall distribution, presence of hotspots, prevalence of subclinical infections, and the population's herd immunity against SARS‐CoV‐2.^21^, ^22^ The point at which the proportion of susceptible individuals falls below the threshold needed for transmission is known as the “herd immunity threshold.”^23^ This can be achieved either by vaccination or natural infection. Herd immunity can be achieved when the level of protective antibodies found in at least 70%–80% in the general population.^24^ However, vaccine distribution and vaccination rate were at the earlier stage at the time of this survey.

COVID‐19 outbreak in Ethiopia and specifically in the Somali Regional State (SRS) of Ethiopia can be followed up in several ways. The first and most important step was to establish diagnostic capacity. The Jigjiga One Health Initiative (JOHI) laboratory at the Jigjiga University (JJU) established RT‐PCR in January 2020. In March the SRS Regional government solicited the purpose of the RT‐PCR for COVID‐19 diagnostic. WHO contributed by funding staffs, the cost and logistics of serological tests. Until December 2020 the JOHI laboratory was the only diagnostic facility in SRS for COVID‐19. Second, serological studies help understanding the true state of the spread of the disease and estimating the seroprevalence of the exposure to the virus. For this purpose, a region wide representative study is needed. Such study provides valuable insights into the extent of COVID‐19 spread within specific communities, contributing to a better understanding of the disease's prevalence and transmission dynamics. This information is essential for guiding public health interventions and vaccination strategies not only in SRS and Ethiopia but also in other countries facing similar challenges.^17^ This study was therefore, designed with the objective of estimating the seroprevalence of antibodies to SARS‐CoV‐2 in the general population of SRS during the COVID‐19 pandemic and risk factors for disease and disease transmission.

## 2 MATERIALS AND METHODS

### 2.1 Description of the area

**Figure 1:**
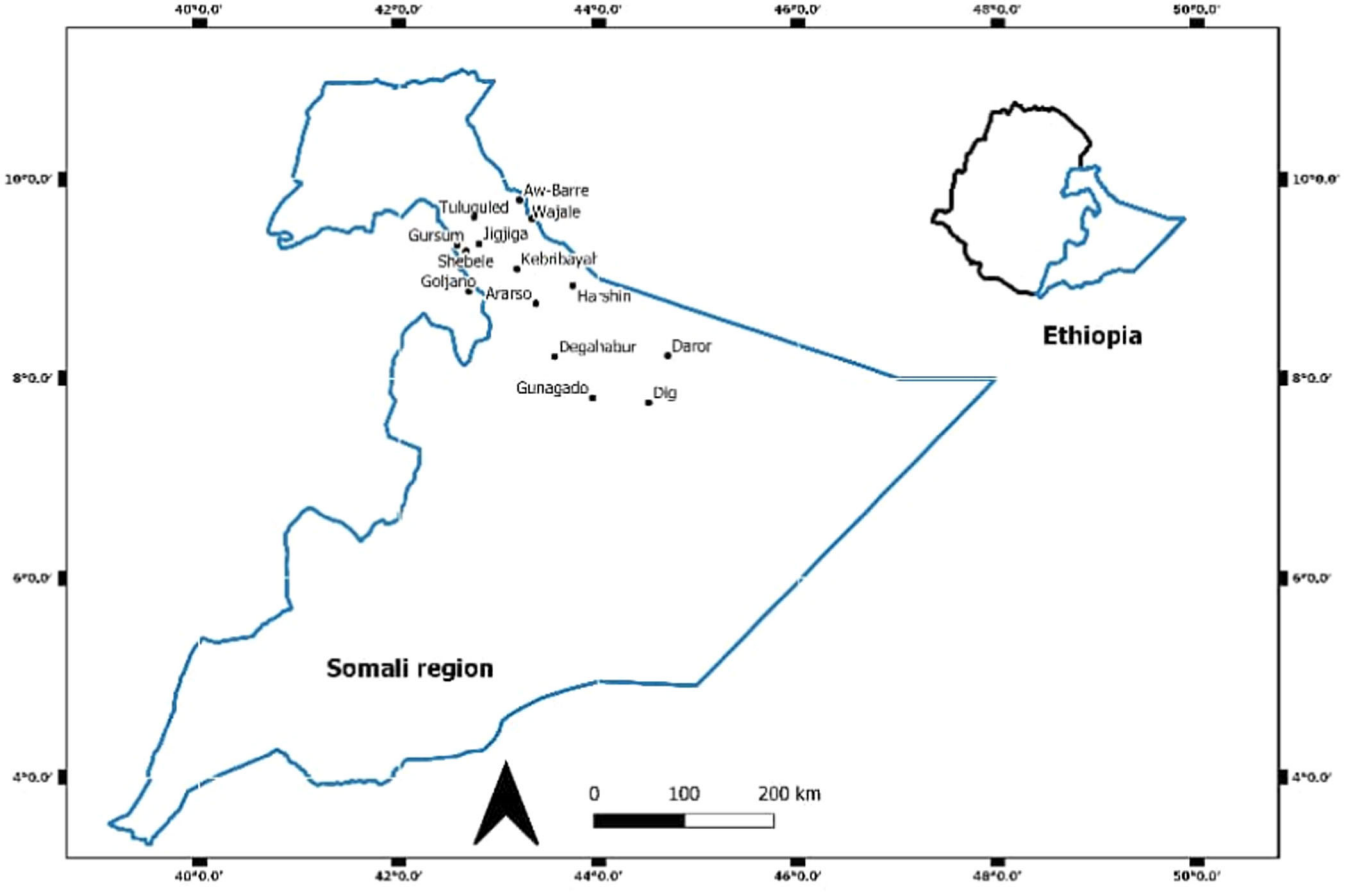
Map of the study area.

This study was carried out in the SRS in eastern Ethiopia, with Jigjiga being its capital, located 635 km from the country's capital, Addis Ababa. The study included 14 districts from two zones (Fafan and Jarar) including Awbare, Goljano, Harshin, Jigjiga, Shebele, Tulliguled, Kebri Beyah, Wajale, Gursum, Degahabur, Daroor, Ararso, Dig, and Gunagado. These study areas were selected based on the geographical location's shared borders with Somaliland in light of the high regional and cross‐border population movement as seen in pastoral contexts, commodity exchange and various social affairs. These sites have therefore a high likelihood of being affected by COVID‐19 (Figure 1).

### 2.2 Study design, population, inclusion, and exclusion criteria

A cross‐sectional study design was conducted from May to June 2021. Samples were collected randomly from participants aged 18 years or older, who showed no clinical symptoms; while those younger than 18 or those who had clinical symptoms were excluded from the study. All participants signed an informed consent before being enrolled in the study.

### 2.3 Sample size determination and sampling procedure

The sample size was determined using the single population proportion formula with an assumed prevalence of COVID‐19 infections of 50%, at 95% confidence interval (CI), 5% marginal error and 10% nonresponse rate, resulting in 820 participants in total to be recruited.

Each district was divided into Kebeles, which are the smallest administrative units. From each district, we randomly selected two kebeles from a provided list of Kebeles. In each Kebele, a geographical central spot was chosen as a sampling unit, based on convenience. The district health officials and health workers invited people from the chosen Kebele to participate in the study by going from house to house. Participants were asked if they wanted to participate, if yes and eligible, they were included. If a participant declined, the study team moved to the next house and person. Recruitment of participants continued at each site until the required sample size was reached in each Kebele.

### 2.4 Data collection procedure

A structured questionnaire was used to collect information related to the sociodemographic characteristics, previous history of SARS‐CoV‐2 PCR testing and/or vaccination and cross‐border travel history in the past 12 months before the study period. Participants were also asked to self‐report whether they had chronic health conditions. In this context chronic health conditions refer to pre‐existing medical conditions that require ongoing medical care and management, included but were not limited to diabetes, heart disease, hypertension and asthma. The questionnaire was carefully reviewed by a research team of Jigjiga University, translated to the local Somali language and pre‐tested.

Trained data collectors were recruited and trained for the study data collection. Regular supervision was done during data collection by the study team. The questionnaires were collected in paper form. The collected data were properly handled, reviewed and checked for completeness and consistency by the supervisors before the data was completed each day.

### 2.5 Blood collection

Each individual enrolled in this study was requested to provide 4–5 mL of venous blood sample for serological analysis. The blood was collected in serum separator tube (SST) via venopuncture by trained nurses and clinical laboratory technicians. Blood samples were taken after verification of compliance with the inclusion criteria and securing informed consent. All personal protection measures were applied before taking the samples. Each sample was labeled with a unique numerical ID (code) to link the questionnaire information and the laboratory results. Blood samples from nearby districts were transported to Jigjiga University Shiek Hassen Yabere Referral Hospital within 3–4 h of collection in a cold box and then serum was separated by centrifugation at 1000*g* for 10 min at room temperature. For specimens collected from distant districts, serum separation was conducted at the district health facility and shipped on ice. About 1 mL of aliquot was transferred into labeled cryovials and stored at −20°C until laboratory analysis.

### 2.6 Laboratory procedure

The serological test was performed using the commercial EUROIMMUN Anti‐SARS‐CoV‐2 ELISA (IgG) kit (Euroimmun), following manufacturer's instructions. This ELISA kit was evaluated and approved by WHO (https://www.finddx.org/covid-19/sarscov2-eval-immuno/). We refer the detail protocol under (https://cdnmedia.eurofins.com/eurofins-us/media/1711222/ei_2606g_a_us_c01_igg_ce.pdf).

Briefly, 100 μL of calibrator, positive control, negative control, and diluted patient sample was added into the individual microplate wells and was incubated for 60 min at +37 ± 1°C. The reagent wells were washed three times with 450 μL of working‐strength wash buffer using a Thermo Scientific well wash instrument (Thermo Fisher Scientific Inc). One hundred microlitres of enzyme conjugate (peroxidase‐labeled anti‐human IgG) was added into each of the microplate wells and incubated for 30 min at +37 ± 1°C. The reagent wells were washed three times with 450 μL of working‐strength wash buffer. One hundred microlitres of chromogen/substrate solution was added into each of the microplate wells and incubated for 30 min at room temperature (+18°C to +25°C). One hundred microlitres of stop solution was added into each of the microplate wells in the same order and at the same speed as the chromogen/substrate solution was introduced. Photometric measurement of the color intensity was made at a wave‐length of 450 nm and a reference wavelength between 620 and 650 nm within 30 min of adding the stop solution using Thermo Scientific Reader instrument (Thermo Fisher Scientific Inc). Before measuring, the microplate was carefully shaken to ensure a homogeneous distribution of the solution. In a positive sample, specific IgG antibodies bound to antigens were detected by an enzyme‐conjugated colorimetric technique. Semiquantitative results were calculated as the ratio between the absorbance of the control or the tested sample and the absorbance of the calibrator. The ratio values of <0.8 were considered negative ≥0.8 to <1.1 as borderline and ≥1.1 as positive. The performance of the current assay was evaluated by Krone et al.^25^ and it was reported 91.2% sensitivity and 96.0% specificity. The assay was authorized by FDA under Emergency Use Authorizations (EUA) and was considered effective in identifying the presence of antibodies to SARS‐CoV‐2.^26^

### 2.7 Data analysis

The outcome variable is the prevalence of serum antibodies against SARS‐CoV‐2 and the seroprevalence rate was calculated as the proportion of participants who tested positive for SARS‐CoV‐2 serum antibodies, among total participants tested. The main independent variables included: demographics, occupational characteristics, and geographical locations. The collected data were entered into Microsoft Excel spreadsheet and exported to Statistical Package STATA (V16.1; STATA Corp) for data cleaning and analysis. Descriptive statistics, such as frequency distribution (*n*) and proportions (%) was computed. Logistic regression for the state of the outcome variable SARS‐CoV‐2 was used. A *p* value of 0.05 or less considered statistically significant.

## 3 RESULTS

### 3.1 Sociodemographic characteristics of study participants

From the total 820 participants surveyed in this study, 477 (58.2%) were male and 343 (41.8%) were female. The majority of the participants were in the 18–34 years' age category (*n* = 581; 70.9%). Most people (60.3%) were married and only 26.6% had education above grade 12. The majority of the participants (*n* = 793; 96.7%) were not vaccinated. Table 1 shows the demographic information of the study participants.

**Table 1:** Selected sociodemographic characteristics of study participants (*n* = 820) for SARS‐CoV‐2 seroprevalence, Somali Region, Ethiopia, 2021.

Demographics	Category	*n* (%)
Sex (*n* = 820)	Female	343 (41.8)
	Male	477 (58.2)
Age in year (*n* = 820)	18–34	581 (70.9)
	10–17	40 (4.90)
	35–49	131 (16.0)
	50–65	54 (6.6)
	>65	13 (1.6)
Family size (*n* = 820)	1–4	341 (41.6)
	5–9	363 (44.3)
	≥10	91 (11.1)
Marital status (*n* = 819)	Single	302 (36.9)
	Married	494 (60.3)
	Divorced	17 (2.10)
	Widowed	6 (0.7)
Education (*n* = 817)	Illiterate	286 (35.0)
	Elementary school	148 (18.1)
	Secondary school	166 (20.3)
	Above Grade 12 including tertiary	217 (26.6)
Occupation (*n* = 817)	Health workers	61 (7.5)
	Education (students, teachers)	176 (21.5)
	Unemployed	218 (26.7)
	Other (driver, merchant, daily laborer, cashier, Government worker)	262 (32.1)
	Refugees	2 (0.2)
	Police/soldiers	38 (4.7)
	Farmers/pastoralists (agriculture)	60 (7.3)
COVID‐19 vaccinated (*n* = 820)	No	793 (96.7)
	Yes	27 (3.3)

### 3.2 Seroprevalence of SARS‐CoV‐2 infection

Out of the 820 collected blood samples 342 people tested positive, thus the crude COVID‐19 prevalence in our study area was 41.7% (95% CI: 33.3%–47.6%).

The highest seroprevalence was observed in Goljano district 70% (*n* = 40) followed by Awbare 55.9% (*n* = 120) and Jigjiga 48% (*n* = 120), while the lowest seroprevalence was observed in Gunagado district 22.5% (*n* = 40) (Table 2).

**Table 2:** Seroprevalence of COVID‐19 in districts of Somali Region.

District	Total tested	Positive (*n*(%))	95% CI
Awbare	120	67 (55.9)	46.8–64.4
Goljano	40	28 (70.0)	54.3–82.1
Harshin	40	14 (35.0)	21.9–50.7
Jigjiga	100	48 (48.0)	38.4–57.7
Shebele	40	18 (45.0)	30.5–60.4
Tulliguled	40	15 (37.5)	24.0–53.2
Kebri Beyah	80	15 (18.8)	11.6–28.8
Wajale	120	50 (41.7)	33.2–50.7
Gursum	40	19 (47.5)	32.7–62.7
Degahabur	40	16 (40.0)	26.1–55.6
Daroor	40	18 (45.0)	30.5–60.4
Ararso	40	10 (25.0)	14.0–40.5
Dig	40	15 (37.5)	24.0–53.2
Gunagado	40	9 (22.5)	12.1–37.9
Overall	820	342 (41.7)	33.3–47.6

Participants with PCR test status, vaccination status, travel history, and history of chronic illness failed to show an association with COVID‐19 seropositivity (Table 3).

**Table 3:** Factors associated with seropositivity to COVID‐19 using univariate analysis.

Factor	Category	Seropositive	OR (95% CI)	*p* Value
Sex	Female	152/343 (44.3)	1	
	Male	190/477 (39.8)	0.79 (0.58–1.07)	.135
Age (year)	18–34	229/581 (39.4)	1	
	10–17	16/40 (40.0)	1.05 (0.53–2.09)	.869
	35–49	72/131 (55.0)	1.90 (1.27–2.83)	.002
	50–65	20/54 (37.0)	0.89 (0.49–1.61)	.706
	>65	4/13 (30.8)	0.74 (0.21–2.52)	.631
Family size	1–4	133/341 (39)	1	
	5–9	159/363 (43.8)	1.17 (0.86–1.60)	.305
	≥10	37/91 (40.6)	0.95 (0.58–1.56)	.861
Marital status	Single	121/302 (40)	1	
	Married	205/494 (41.5)	1.06 (0.78–1.44)	.707
	Divorced	8/17 (47.0)	1.36 (0.49–3.74)	.543
	Widowed	3/6 (50.0)	1.69 (0.31–9.27)	.541
Education	Illiterate	124/286 (43.3)	1	
	Elementary school	62/148 (41.9)	0.91 (0.59–1.38)	.665
	Secondary school	65/166 (39.1)	0.70 (0.45–1.08)	.108
	Above Grade 12 including tertiary	91/217 (41.9)	0.79 (0.53–1.17)	.246
Occupation	Health workers	27/61 (44.3)	1	
	Education (students, teachers)	73/176 (41.5)	0.83 (0.44–1.58)	.583
	Unemployed	94/218 (43.1)	1.12 (0.60–2.08)	.707
	Other (driver, merchant, daily laborer, cashier, Gov worker)	104/262 (39.7)	0.88 (0.48–1.60)	.678
	Refugees	1/2 (50.0)	0.76 (0.04–13.3)	.856
	Police/soldiers	14/38 (36.8)	0.79 (0.29–2.15)	.654
	Farmers/pastoralists (agriculture)	28/60 (46.7)	1.17 (0.53–2.55)	.690
Chronic health condition	No	316/768 (41.1)	1	
	Yes	20/39 (51.3)	1.54 (0.78‐3.05)	.206
Travel history in last 12 months	No	292/705 (41.4)	1	
	Yes	48/113 (42.5)	1.06 (0.60–1.85)	.829
COVID‐19 vaccinated	No	326/793 (41.1)	1	
	Yes	16/27 (59.2)	2.14 (0.90–5.10)	.083
PCR test in last 6 months	Negative	22/50 (44.0)		
	Positive	1/3 (33.3)	0.47 (0.03–6.81)	.587

Among the demographic characteristics, sex, family size, marital status, level of education, and occupation were not associated with positive status. In contrast, age group of 35–49 years old was significantly associated with a higher seropositivity as compared with other age groups (OR = 1.90; *p* = .002).

## 4 DISCUSSION

Seroprevalence studies provide information on the number of persons who have been exposed to the virus. Seroepidemiological studies also can give us an estimation of the proportion of the population still susceptible to the infection as it is assumed that antibodies provide immunity.^17^ It is also important in delivering focused preventative and control techniques to reduce transmission and catastrophic effects. Our study was the first large‐scale community‐based sero‐surveillance of COVID‐19 conducted in SRS. It allowed gaining an insight on prevalence among the community understand the distribution of the disease exposure among the districts in light of remoteness of sites, poor health surveillance and delivery services, highly mobile population, and cross‐border movement. We acknowledge that our data is derived from a specific area, SRS. However, we believe that the aforementioned unique characteristics of SRS provide valuable COVID‐19 information that may have broader implications.

The first case of COVID‐19 in Somali Region was detected on 25 April 2020, from a 48‐year‐old man with no travel history presenting himself at Yilak Specialized Clinic in Jigjiga city (Personal Communication). The present study showed that the overall seroprevalence of COVID‐19 in the region was 41.7%, more than 11 months after the first case was detected in the region. This finding was the highest ever reported seroprevalence in Ethiopia.^27^, ^28^, ^29^, ^30^, ^31^ This variation in seroprevalence is likely due to the different time of the conducted survey within the pandemic timeline. Sero‐surveys were conducted when the epidemic was in its earlier stage in Ethiopia.^23^, ^24^, ^27^, ^28^, ^29^ In contrast, our study was conducted at a later stage (May to June 2021). As a result, we expected a high proportion of the population who would have been exposed to the virus and developed antibodies. Moreover, the difference was also likely attributable to methodological differences, including target group, test kit type, and sample size.

The highest prevalence was observed in Goljano district, followed by Awbare, Jigjiga, and Gursum districts. The highest prevalence rate in Goljano district may be explained by the lack of public health measures and low disease awareness. The district lacks electric power and internet access. The lack of these services might have a big impact on the level of received disease awareness and the use of preventive practices toward COVID‐19 pandemic. Energy poverty in Ethiopia was challenged by the COVID‐19 pandemic in terms of education systems and sharing information about the disease to create awareness using online information sharing technology.^32^

The district of Awbare and Tog Wajale are close to the Somaliland border, where there has been a lot of cross‐border movement. Due to the fact that these border towns serve as major commercial hubs and exchange points for various commodities, they are crucial both demographically and economically for Ethiopia and Somaliland. During the COVID‐19 pandemic, these may have contributed to the increased risk of the virus spreading across the districts. One of the likely causes of the COVID‐19 virus' quick proliferation in Ethiopia was reported to be the constant flow of immigrants and the inadequate border protection.^33^ Likewise similar high prevalence rates in border towns and cities were reported in Ruili City of China‐Myanmar border^34^ and in Baja California of Mexico‐USA border.^35^

High seroprevalence rate reported in Jigjiga district might be due to its large population size and relatively urbanization, where there is considerable overcrowding. Higher seroprevalence levels were observed in cities with large populations in Colombia.^36^ High population densities imply frequent face‐to‐face interactions, crowding, and wide‐ranging social networks. Cities are plausible protagonists in the spread of the COVID‐19 pandemic.^37^ Seroprevalence result in Jigjiga obtained in this study was considerably higher than the seroprevalence of 3.3% reported by the national population‐based serosurvey, conducted in the same city in June to July 2020.^29^ The rapid increase of the seroprevalence from 3.3% to 48% in 1 year shows the rapid dynamics of the pandemic. Gunagado district reported the lowest COVID‐19 prevalence rate. People in that district lived in sparsely inhabited villages and were at a considerable distance from any urban areas. This is in line with a population‐based seroepidemiological study conducted in South India, where rural districts had a lower seroprevalence of COVID‐19 infection.^17^

Among all assessed risk factors, only the age group including 35–49 years old showed statistically significant association with a higher seropositivity as compared with other age groups. Prevalence increased to adolescence and remained fairly stable at older ages. Similar findings were reported in South India^17^ and in Spain.^9^ Age has likely a profound influence on mobility and varies across cultures. Hence, the susceptibility to infection can be attributed to the function of mobility rather than age per se. Mobility though did not affect seroprevalence in our study. Several studies investigated mobility and found a positive association.^38^ Possible explanation may be the low variation of mobility due to low restrictions across the study participants and the location of travels.

Our study found that vaccination status against COVID‐19 was not substantially related with COVID‐19 seroprevalence, although the majority of participants were not vaccinated. At the time the study was conducted, distribution of COVID‐19 vaccine was in its earlier stage.

Only 39 (4.76%) of the patients had reported known underlying medical conditions and there was no association with seropositivity. Although underlying chronic medical conditions have been associated with an increased risk of COVID‐19‐related hospitalization and increased mortality,^39^, ^40^, ^41^ the general population may be equally susceptible to the infection.^17^ Moreover, it could be due to the data collection method in which the assessment for the presence of chronic medical conditions was based only on patients' self‐report only, introducing possible reporting bias.

Other independent factors such as sex, family size, marital status, education, occupation, travel history for the last 12 months, and PCR test in the last 6 months were not significantly associated with COVID‐19 seropositivity.

This study had some limitations. When assessing risk factors, we did not take into account variables such as community preventive measures practices, and mindset. It would not have had a substantial impact on the study's results, and the data would help local public health officers to identify risk factors and offer information on COVID‐19 dispersion in the population, including rural places. Additionally, vaccinated individuals were included in the analysis. We acknowledge that the inclusion of vaccinated individuals in our seropositivity analysis may introduce bias. However, this potential bias might not affect the outcome due to the low percentage of vaccinated individuals included in the analysis.

## 5 CONCLUSION

The present study provides a region wide estimate of SARS‐CoV‐2 prevalence and hence geographical spread of the disease in the Somali Region. The high prevalence of COVID‐19 combined with the low vaccination rate indicated that a large proportion of the population in SRS had been infected since the beginning of 2020. However, herd immunity was not achieved since half of the population was still susceptible. As a result, it is vital to promote and strengthen the COVID‐19 preventative measures to limit transmissions within the community and access to immunization is still of paramount importance. Moreover, the key findings from the present study will have implications for shaping future epidemic control.

## AUTHOR CONTRIBUTIONS

Solomon Yared and Tsegalem Abera conceived research idea, study design, conducted laboratory testing, and conducted manuscript drafting. Seid Mohammed Ali and Rea Tschopp conceived research idea and conducted manuscript drafting. Jakob Zinsstag and Rea Tschopp conducted study design. Jan Hattendorf and Rea Tschopp conducted statistical analysis. Abdifatah Muktar Muhummed, Mohammed Ibrahim, and Abdullahi Hassan revised the content of the manuscript, supervised and assisted the study. All authors contributed to the interpretation of the findings. All authors critically revised the paper for intellectual content and approved the final version of the manuscript.

## CONFLICT OF INTERESTS STATEMENT

The authors declare no conflict of interest.

## ETHICS STATEMENT

This study was reviewed by the Jigjiga University Internal Review Board (JJU/REC045/2020).

## Data Availability

The data that support the findings of this study are available from the corresponding author upon reasonable request.
